# A Novel Neuroprotective Mechanism for Lithium That Prevents Association of the p75^NTR^-Sortilin Receptor Complex and Attenuates proNGF-Induced Neuronal Death *In Vitro* and *In Vivo*

**DOI:** 10.1523/ENEURO.0257-17.2017

**Published:** 2018-01-17

**Authors:** Shayri G. Greenwood, Laura Montroull, Marta Volosin, Helen E. Scharfman, Kenneth K. Teng, Matthew Light, Risa Torkin, Fredrick Maxfield, Barbara L. Hempstead, Wilma J. Friedman

**Affiliations:** 1 Department of Biological Science, Rutgers University, Newark, NJ 07102; 2 Nathan Kline Institute, Orangeburg, NY 10962; 3 Department of Medicine, Weill Cornell Medical College, New York, NY

**Keywords:** apoptosis, neuroprotection, neurotrophins, p75, proNGF, seizures

## Abstract

Neurotrophins play critical roles in the survival, maintenance and death of neurons. In particular, proneurotrophins have been shown to mediate cell death following brain injury induced by status epilepticus (SE) in rats. Previous studies have shown that pilocarpine-induced seizures lead to increased levels of proNGF, which binds to the p75^NTR^-sortilin receptor complex to elicit apoptosis. A screen to identify compounds that block proNGF binding and uptake into cells expressing p75 and sortilin identified lithium citrate as a potential inhibitor of proNGF and p75^NTR^-mediated cell death. In this study, we demonstrate that low, submicromolar doses of lithium citrate effectively inhibited proNGF-induced cell death in cultured neurons and protected hippocampal neurons following pilocarpine-induced SE *in vivo*. We analyzed specific mechanisms by which lithium citrate afforded neuroprotection and determined that lithium citrate prevented the association and internalization of the p75^NTR^-sortilin receptor complex. Our results demonstrate a novel mechanism by which low-dose treatments of lithium citrate are effective in attenuating p75^NTR^-mediated cell death *in vitro* and *in vivo*.

## Significance Statement

Neuronal death occurs after prolonged severe seizures and is partially due to the induction of proNGF and its p75 neurotrophin receptor. The p75^NTR^ utilizes a coreceptor, sortilin, to bind proNGF and promote apoptotic signaling. We show here that submicromolar concentrations of lithium citrate prevented p75^NTR^-mediated neuronal death by impairing the formation and internalization of the coreceptor complex. Although lithium has many neuroprotective functions that occur at millimolar concentrations, we demonstrate a novel mechanism for lithium citrate to afford neuroprotection from seizure-induced death at submicromolar doses.

## Introduction

The neurotrophin growth factors regulate many aspects of neuronal function, including cell survival and death. Neurotrophins are initially synthesized as precursor proneurotrophins and are cleaved to generate their C-terminal mature forms, which bind to the Trk family of receptor tyrosine kinases to enhance neuronal survival and differentiation (Huang and Reichardt, 2003; Reichardt, 2006). In contrast, proneurotrophins bind with high affinity to a receptor complex comprised of p75^NTR^ and sortilin, which can initiate apoptotic signaling (Lee et al., 2001; Nykjaer et al., 2004). Following injury, proneurotrophins and p75^NTR^ are upregulated and can play a significant role in promoting neuronal cell death. Previous studies have demonstrated that prolonged severe seizures, induced by either pilocarpine or kainic acid, promote neuronal death in rats mediated by p75^NTR^ (Troy et al., 2002). Seizures also elevate levels of proNGF, a potent ligand for the activation of p75^NTR^-mediated cell death (Volosin et al., 2008), and prevent its cleavage by MMP7, leading to increased levels of intact proNGF in the extracellular environment (Le and Friedman, 2012). ProNGF induces neuronal death by interacting with a receptor complex consisting of p75^NTR^ and sortilin (Nykjaer et al., 2004; Hempstead, 2009). Moreover, the two receptors can be recruited to the cell surface by inflammatory cytokines, thereby increasing vulnerability to proNGF after brain injury (Choi and Friedman, 2014).

Recently, we performed a drug screen to identify compounds that block the binding of proNGF to cells expressing sortilin and p75^NTR^, and lithium citrate was among these compounds. Lithium ion can inhibit apoptosis by a variety of different mechanisms (Wada et al., 2005), including increasing Akt activity, by phosphorylating and inactivating GSK3β (Tajes et al., 2009; Pasquali et al., 2010), and promoting autophagy (Motoi et al., 2014; Del Grosso et al., 2016; Liu et al., 2017). Chronic treatment with lithium has also been shown to upregulate BDNF expression in the brain (Fukumoto et al., 2001) and retina (Wu et al., 2014), which provides another potential neuroprotective mechanism for lithium ion. However, since the drug screen identified lithium citrate by preventing the binding and uptake of proNGF to its p75^NTR^-sortilin receptor complex, another potential target for lithium ion could be in altering this receptor complex.

In a rat experimental model of temporal lobe epilepsy (TLE), status epilepticus (SE) induced by pilocarpine causes a defined pattern of damage in the hippocampus with severe loss of neurons in the CA1 region as well as the hilus/dentate gyrus region (Turski et al., 1984, 1989). Previous studies have demonstrated that much of the seizure-induced neuronal loss is due to the upregulation of proNGF and p75^NTR^ (Roux et al., 1999; Troy et al., 2002; Volosin et al., 2008), and blocking proNGF-p75^NTR^ signaling, using function-blocking polyclonal antibodies to the prodomain of proNGF, attenuates hippocampal cell death following seizures (Volosin et al., 2008; Song et al., 2010). Therefore, in these studies, we investigated the mechanisms by which lithium citrate affords neuroprotection from proNGF induced cell death, and tested the efficacy of lithium citrate in preventing p75^NTR^-mediated cell death following pilocarpine-induced seizures. We demonstrate that low doses of lithium, well below the dose used for the standard lithium-pilocarpine model of epilepsy (Jope et al., 1986) and below the dose needed to phosphorylate GSK3β or induce BDNF (Wada et al., 2005), decreased the association and internalization of the p75^NTR^-sortilin receptor complex, and prevented proNGF-induced neuronal apoptosis in culture and *in vivo*.

## Materials and Methods

### Alexa Fluor 594 labeling of proNGF

Twenty micrograms of purified human proNGF, prepared in SF9 cells as described (Feng et al., 2010), was added to 10 µl of PBS and 7 µl of reconstituted Alexa Fluor 594 (excitation 590 nM, emission 617 nM; Invitrogen) according to the manufacturer’s protocol (microscale protein labeling kit A30008) and incubated at room temperature, in the dark, for 15 min. Tris, pH 8.0, was added to obtain a final concentration of 50 mM to quench the labeling reaction. Labeled proNGF was then extensively dialyzed using PBS, pH 7.4, at 4°C in the dark. Alexa Fluor-labeled proNGF was used within 48 h. HT-1080 cells that stably expressed p75 and sortilin (generated as described; Feng et al., 2010), were cultured in 384 well flat bottom plates (CellBind) for 24 h. Cells were treated with one of 2560 compounds from the SpecPlus Collection (MicroSource Discovery Systems) at a concentration of 10 µM, or 20 µM neurotensin, or diluent control, using an automated robotics system, followed by addition of 200 nM Alexa Fluor-proNGF. Cells were incubated for 18 h, then rinsed with PBS, and fixed with 4% paraformaldehyde for 10 min, rinsed three times with PBS, and then counterstained using Hoechst at a final concentration of 10 µg/ml for 2 h at room temperature. Cells were analyzed for uptake of Alexa Fluor 594-conjugated proNGF using a Discovery 1 automatic fluorescence microscope from Molecular Devices as described previously (Pipalia et al., 2006).

Images were acquired with a Photometrics Cool Snap HQ camera and analyzed using Metapmorph Discovery 1 image analysis software. To correct for shading, an image was created by averaging all of the images from a plate and smoothing the averaged image with a low pass filter. Thresholding was performed, using a low threshold to include all areas occupied by cells. The outlines of cells were selected, as were the outlines of nuclei, assessed by Hoecht staining. The number of pixels in the area of the cell within two nuclear diameters of the nuclei were calculated, and the average proNGF intensity was calculated as the total intensity above the low threshold/number of pixels above low threshold (modified from Pipalia et al., 2006).

Normalized values were obtained by dividing the values in the presence of each compound by the values obtained in the presence of solvent control in each plate. All compounds were tested in replicates of eight. Compounds from the SpecPlus Collection (MicroSource Discovery Systems) were assayed in cells that expressed p75^NTR^ and sortilin. The compounds in the collection are primarily Food and Drug Administration-approved compounds or natural products.

### Primary hippocampal neuronal cultures

All animal studies were conducted using the National Institutes of Health guidelines for the ethical treatment of animals with approval of the Rutgers Institutional Animal Care and Facilities Committee.

Rat hippocampi were dissected from embryonic day 18 animals and dissociated as previously described (Friedman, 2010). Dissociated neurons were then plated on poly-d-lysine (0.1 mg/ml)-coated dishes maintained in serum free media. The media consisted of 1:1 MEM and F12, with glucose (6 mg/ml), insulin (2.5 mg/ml), putrescine (60 µM), progesterone (20 nM), transferrin (100 µg/ml), selenium (30 nM), penicillin (0.5 U/ml), and streptomycin (0.5 µg/ml). Neuronal cultures were maintained in media for 5 d before treatment with proNGF and lithium citrate.

### Cell culture treatments and survival assay

Lithium citrate doses in the nanomolar and micromolar ranges did not show toxicity in prescreen testing and was used here at doses ranging from 10 nM to 100 µM. Following proNGF (2-5 ng/ml) and lithium citrate treatment, hippocampal neurons were lysed and healthy nuclei were counted using a hemocytometer to assess cell viability (Friedman, 2010). To distinguish between nuclei of healthy cells and those of dead cells, pyknotic and irregular membrane shapes common to cells dying via apoptosis were assessed and excluded. Cell counts were performed in triplicate.

6-Bromoindirubin-3’-oxime (BIO) was purchased from Sigma and dissolved in DMSO for a stock concentration of 3 mM.

For Western blot analysis, hippocampal neurons plated in six-well dishes (1 × 10^6^ cells/well) were treated as indicated, washed in sterile PBS (pH 7.4) and lysed in buffer containing 120 mM Tris, 2% SDS, 10% glycerol and protease inhibitors. Equal amounts of protein were subjected to PAGE, transferred to nitrocellulose membrane and blocked with 5% nonfat milk. Blots were incubated in primary antibodies to GSK3β, p-GSK3β, BDNF, and actin overnight. After washing three times with TBST for 15 min each, the blots were incubated with appropriate secondary antibodies for 1 h at room temperature. The membrane was washed three times with TBST before being visualized using either ECL (Pierce) or scanned with the Odyssey infrared imaging system (LI-COR Bioscience). To ensure equal protein levels, blots were stained with Ponceau and reprobed with actin. All analyses were performed at least three times in independent experiments.

### Pilocarpine induced seizures

Adult male Sprague Dawley rats (250-350 g) were pretreated with methyl-scopolamine (1 mg/kg, s.c.; Sigma) to prevent peripheral effects 30 min before giving pilocarpine (350-380 mg/kg) to induce SE. One hour following the onset of SE (Racine scale stage 5 behavior), animals were treated with diazepam (10 mg/kg) and phenytoin (50 mg/kg) to reduce the seizures. Control animals received the same treatments except they received saline instead of pilocarpine. Animals were given Hartmann’s solution (130 mM NaCl, 4 mM KCl, 3 mM CaCl, and 28 mM lactate; 1 ml/100 g) daily until they were capable of eating and drinking ad libitum and monitored for 3 d.

To assess the effects of lithium citrate on neuronal death *in vivo*, animals were treated with lithium citrate (6 mg/kg, s.c.) 30 min before receiving pilocarpine or saline. To assess whether lithium could provide effective neuroprotection if given after the seizures, a different cohort of animals was given lithium citrate after the seizures were stopped with diazepam and phenytoin. In each experiment, animals were designated as controls, pilocarpine, pilocarpine + lithium, and lithium alone. Rats treated with lithium citrate were given injections every 12 h for 3 d to maintain relatively constant levels of lithium (Malhi and Tanious, 2011). Levels of lithium ion that entered the brain were measured in CSF collected at the time of euthanasia.

### Immunohistochemistry

Animals were anesthetized with ketamine/xylazine and perfused transcardially with saline followed by 4% paraformaldehyde. The brains were removed and postfixed in 4% paraformaldehyde for 2 h and cyroprotected in 30% sucrose overnight. Sections (12 µm) were cut on a cryostat (Leica) and mounted onto charged slides. Sections were blocked in PBS/5% BSA and permeabilized with PBS/0.3% Triton X-100, and then exposed to primary antibodies overnight at 4°C in PBS/1% BSA. Slides were then washed three times in PBS, exposed to secondary antibodies coupled to different fluorophores at room temperature for 1 h in the dark. Sections were washed again three times, with 4’,6’-diamidino-2-phenylindole (DAPI; Sigma; 1:10,000) present in the final wash. Sections were coverslipped with antifading medium (ProLong Gold; Invitrogen) and analyzed by fluorescence microscopy (Nikon). Primary antibodies used are as follows: anti-p75 (1:500; R&D Systems, RRID:AB_2298561) and anti-cleaved caspase-3 (CC3; 1:1000; Cell Signaling Technology, RRID:AB_2069869).

### Fluoro-Jade C labeling

The number of dying neurons following pilocarpine induced seizures was assessed by labeling with Fluoro-Jade C (Millipore) according to the manufacturer’s protocol. Sections were then immunostained with anti-p75^NTR^.

### Coimmunoprecipitation and Western blotting

Cultured hippocampal neurons were treated with lithium citrate for 30 min followed by a 30-min treatment with proNGF and compared with neurons treated with proNGF alone, lithium citrate alone and untreated control neurons. Cells were harvested in a buffer containing 0.6 M octylglucoside, 10% Triton X-100, 10× TNE with a phosphatase inhibitor cocktail tablet (Roche). Whole-cell lysates were precleared with protein G-Sepharose beads (Pierce) at 4°C for 60 min. The cleared lysates were incubated overnight at 4°C with α-p75^NTR^ (192 IgG, Millipore) followed by a 2-h incubation at 4°C with protein G-Sepharose beads. Finally, the beads were washed five times with the buffer described above, eluted by boiling in loading buffer for SDS-PAGE. Equal amounts of protein were separated by 8% PAGE, transferred to nitrocellulose membranes, and probed for sortilin (diluted 1:500, BD Sciences) and p75^NTR^ (diluted 1:500, Cell Signaling). All Western blot analyses were performed at least three times with samples from independent experiments.

For the *in vivo* experiments, hippocampi were dissected 3 d after the seizures and homogenized in RIPA buffer. Lysates were cleared with protein G-Sepharose and incubated overnight with anti-sortilin (BD Science), followed by a 2-h incubation with protein G-Sepharose beads. Samples were analyzed by Western blotting for p75^NTR^ (Millipore).

### Biotinylation assays

Cell surface receptor biotinylation assays were performed using cultured hippocampal neurons. Cultures were rinsed with PBS and subsequently washed with PBS containing magnesium chloride and calcium chloride. Cultures were then biotinylated with sulfo-NHS-S-S-biotin (Pierce). Cells were rinsed with 100 nM glycine to quench remaining biotin, and were then incubated at 37°C for 10 min in media alone (control) or in media containing proNGF (3 ng/ml), lithium citrate (100 nM), or proNGF + lithium citrate. Remaining cell surface biotin was cleaved with 50 mM glutathione, 75 mM NaCl, 75 mM NaOH, 0.01 g/ml BSA, and 10 mM EDTA, and cells were lysed in RIPA buffer with protease inhibitors. Biotinylated proteins were precipitated with streptavidin beads to pull down internalized receptors, followed by immunoblotting for p75^NTR^ and sortilin. Each experiment was repeated at least three times.

### Quantification and statistical analysis

For quantification of immunostaining analysis, every 8th section throughout the hippocampus was processed for p75^NTR^/CC3 double immunocytochemistry. Double-labeled cells from the hilus and CA1 regions, areas susceptible to pilocarpine-induced damage, were counted on both sides of the hippocampus. Adjacent sections were taken for analysis of p75^NTR^/fluorojade double-labeled cells in the hilus and CA1 regions of the hippocampus. The number of labeled cells is expressed as percentage control relative to the number of labeled cells in control brains. Statistical analysis was performed using ANOVA with Tukey’s *post hoc* analysis, and *p* < 0.05 was considered significant.

For quantification of immunoprecipitation and biotinylation analysis, bands were quantified densitometrically and are shown as the mean of three independent experiments. Statistical analysis was performed using ANOVA with Tukey’s *post hoc* analysis and *p* < 0.05 was considered significant.

## Results

### Screen to identify inhibitors of proNGF binding and uptake to p75^NTR^-sortilin expressing cells

We performed a drug screen to identify compounds that blocked the binding and internalization of fluorescently-labeled (Alexa Fluor) proNGF using cells stably expressing p75^NTR^ and sortilin. Stable clones of human fibrosarcoma HT-1080 cells expressing p75^NTR^, sortilin, or both receptors were generated, and the receptor expression was confirmed by Western blot analysis. Our prior studies using these conditions documented that coexpression of p75^NTR^ and sortilin led to enhanced uptake of labeled proNGF, as compared to cells expressing comparable levels of p75^NTR^ or sortilin alone (Feng et al., 2010). Binding and uptake of Alexa Fluor-proNGF was inhibited by >90% on concomitant treatment with 10 µM neurotension, consistent with prior studies in which neurotensin impaired the crosslinking of proNGF to p75/sortilin complexes, and impaired proNGF-induced apoptosis (Nykjaer et al., 2004). Compounds from a commercially available library were screened for reduction in proNGF-uptake. Drugs (at 10 µM concentration) that exhibited a 66% reduction in uptake, and resulted in >85% viability as compared to control, were considered potential antagonists. Fifteen compounds which met these criteria, and have been used in humans are listed in Table 1. Lithium citrate was among the 15 candidate compounds identified, inhibiting proNGF uptake by 66.4% as compared to diluent control, and exhibited no significant toxicity (<5% cell loss as compared to control). Therefore, lithium citrate was further evaluated using cultured neurons.

**Table 1.** List of compounds from the screen of the SpecPlus Collection that blocked proNGF uptake

Compound
Levodopa
2’,2’-Bisepigallocatechin monogallate
Meclizine hydrochloride
Suramin
Atorvastatin calcium
Miglitol
Acetyl tyrosine ethyl ester
Prazosin hydrochloride
Lovastatin
Glyburide
Quercetin pentamethyl ether
Perindopril erbumine
Almotriptan
Oxaprozin
Lithium citrate

Fifteen compounds were identified from the 2560 screened that that exhibited a 66% reduction in proNGF uptake at 10 μM concentration and resulted in >85% viability as compared to control.

### Low dose of lithium protects hippocampal neurons from proNGF-induced death in vitro

**Figure 1.**
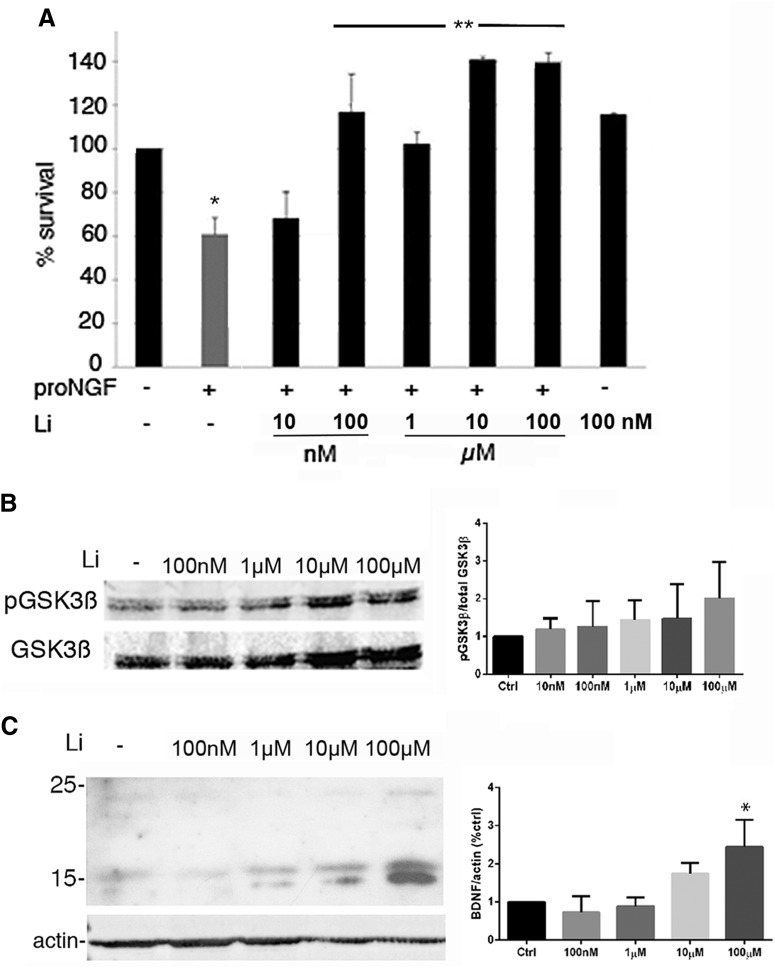
Dose-dependent effects of lithium citrate on hippocampal neurons. ***A***, E18 hippocampal neurons were cultured for 5 d and treated overnight with or without proNGF (3 ng/ml) alone or in the presence of different doses of lithium citrate. Cells were then lysed and healthy nuclei of surviving neurons were counted; 100 nM lithium citrate was sufficient to protect neurons from proNGF. ***B***, Dose response for lithium citrate to induce phosphorylation of GSK3β *in vitro*. Cultured hippocampal neurons were treated with the indicated doses of lithium citrate and analyzed by Western blotting for p-GSK3β. Blots were stripped and reprobed for total GSK3β. Quantification of four independent experiments is shown. ***C***, Dose response for lithium citrate to induce BDNF expression. Cultured hippocampal neurons were treated with the indicated doses of lithium citrate and analyzed by Western blotting for BDNF. Blots were stripped and reprobed for actin. Quantification of three independent experiments is shown, * significant at *p* < 0.05 by ANOVA.

To analyze mechanisms by which lithium citrate could protect hippocampal neurons from proNGF-induced death, cultured neurons were treated with proNGF with or without lithium citrate pretreatment. A dose-response analysis demonstrated that lithium citrate protected neurons from proNGF-induced death, with the lowest protective dose of 100 nM (Fig. 1*A*). Lithium ion is known to be neuroprotective by a variety of mechanisms (Wada et al., 2005; Young, 2009), including via phosphorylation and inactivation of GSK3β, therefore we investigated whether the doses at which lithium citrate prevented proNGF-induced death were sufficient to phosphorylate GSK3β. Interestingly, the lowest protective doses of 100 nM and 1 µM were insufficient to phosphorylate GSK3β (Fig. 1*B*). In addition, a different method of inactivating GSK3β using BIO (Meijer et al., 2003), rather than lithium citrate was unable to protect hippocampal neurons from proNGF-induced apoptosis (not shown). These data indicate that the neuroprotective actions of lithium citrate were not mediated by GSK3β phosphorylation and inactivation. Additionally, chronic treatment with lithium has also been shown to upregulate BDNF expression in the brain (Fukumoto et al., 2001) and retina (Wu et al., 2014), which provides another potential neuroprotective mechanism for lithium. Therefore, we compared the dose of lithium citrate that was required for BDNF induction with the dose that protected hippocampal neurons from proNGF-induced apoptosis. Robust induction of BDNF was observed at 100 µM, a dose at least 1000-fold higher than the dose required for protection from proNGF (Fig. 1*C*). These data indicate that the protection afforded by lithium citrate was due neither to phosphorylation of GSK3β nor to induction of BDNF, and might represent a novel mechanism.

### Lithium citrate prevents the association and internalization of the p75^NTR^-sortilin receptor complex

**Figure 2.**
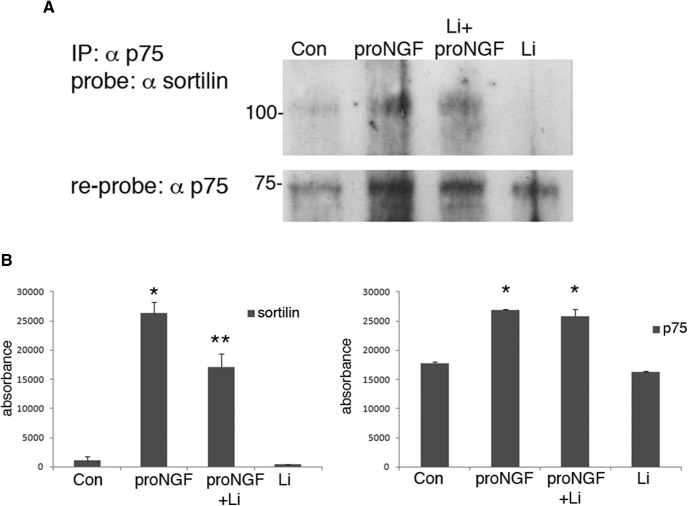
Lithium citrate decreases the association between p75^NTR^ and sortilin. E18 hippocampal neurons were cultured for 5 d and treated with vehicle or proNGF (3 ng/ml) for 30 min, with or without a 30-min pretreatment with 100 nM lithium citrate. Cells were lysed, immunoprecipitated with anti-p75^NTR^, and probed with anti-sortilin. Blots were reprobed with anti-p75^NTR^. ***A***, Representative blot showing co-IP of p75^NTR^ and sortilin and reprob for p75^NTR^. ***B***, Densitometric quantification of sortilin and p75^NTR^ bands from three independent experiments; *different from control *p* < 0.05, **different from proNGF *p* < 0.05.

The initial identification of lithium citrate as being protective from proNGF-induced apoptosis was based on a screen that blocked binding to the p75^NTR^-sortilin receptor complex and subsequent internalization, therefore we assessed whether lithium citrate could act at the level of the membrane receptors to prevent proNGF actions by interfering with the formation of the p75^NTR^-sortilin receptor complex, or internalization of the receptors. Cultured hippocampal neurons were treated for 30 min with proNGF with or without a 30-min pretreatment with 100 nM lithium citrate and compared to untreated neurons or treatment with lithium citrate alone. Cell lysates were immunoprecipitated with anti-p75^NTR^, probed for sortilin and reprobed for p75^NTR^. Levels of p75^NTR^ were increased by proNGF treatment, which was not affected by the lithium citrate pretreatment, however the amount of coimmunoprecipitated sortilin was significantly reduced by the lithium citrate pretreatment (Fig. 2), suggesting that lithium citrate attenuated the association between sortilin and p75^NTR^.

**Figure 3.**
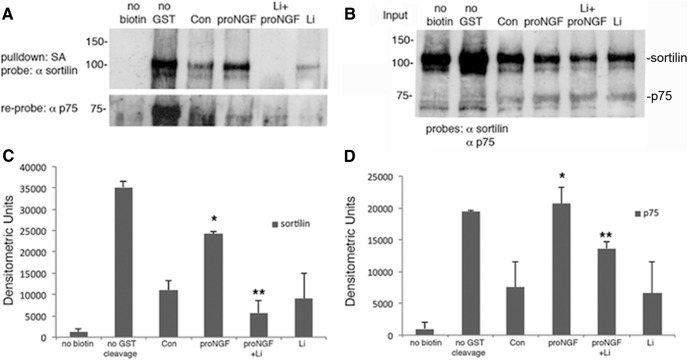
Lithium citrate decreases the internalization of p75^NTR^ and sortilin. ***A***, E18 hippocampal neurons were cultured for 5 d, biotinylated, and treated for 10 min with proNGF. After stripping remaining surface biotin with glutathione, streptavidin was used to pull down the internalized, biotinylated proteins, which were analyzed by Western blotting for sortilin and p75^NTR^. ***B***, Lysates before streptavidin pulldown were also analyzed by Western blotting for sortilin and p75^NTR^ to assess the input. Densitometric quantification of biotinylated sortilin (***C***) and p75^NTR^ (***D***) from three independent experiments; *different from control at *p* < 0.05, **different from proNGF at *p* < 0.05.

Treatment with proNGF elicits internalization of the p75^NTR^-sortilin receptor complex, therefore we also investigated whether lithium citrate treatment could affect receptor internalization. Cell surface biotinylation experiments investigated the internalization of the receptors after proNGF treatment. Cultured hippocampal neurons were biotinylated and then incubated with proNGF for 10 min with or without pretreatment with lithium citrate. Surface biotin was then stripped off and streptavidin was used to pull down internalized biotinylated proteins, which were probed for p75^NTR^ (Fig. 3*A*,*D*) and sortilin (Fig. 3*A*,*C*). Pretreatment with 100 nM lithium citrate significantly reduced the internalization of sortilin and p75^NTR^ (Fig. 3). Thus, the low dose of lithium citrate (100 nM) that was protective from proNGF-induced neuronal death attenuated the association and internalization of the p75^NTR^-sortilin receptor complex.

### Lithium citrate protects hippocampal neurons from pilocarpine-induced neuronal loss

**Figure 4.**
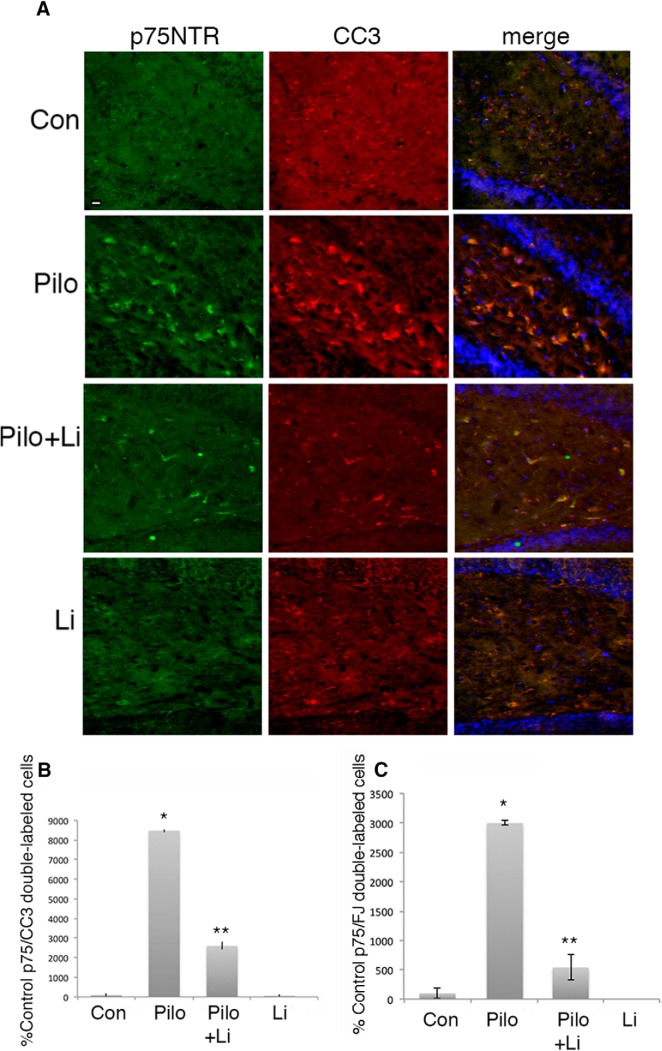
Lithium citrate pretreatment prevents neuronal death in the hilus following seizures *in vivo*. Adult male rats were subjected to pilocarpine-induced seizures with or without a 30-min pretreatment with lithium citrate (6 mg/kg) and injections twice daily for 3 d. ***A***, Sections through the hilus showing double labeling with anti-p75^NTR^ and anti-CC3. ***B***, Quantification of p75^NTR^/CC3 double-labeled cells in the hilus with the different treatments. Cells were counted in every 8^th^ section through the hippocampus. ***C***, Quantification of p75^NTR^/fluorojade-labeled cells in the hilus with the indicated treatments. Scale bars: 10 µm. Quantification in ***B***, ***C*** is expressed as percentage control values which are set at 100%. * different from control at *p* < 0.05, ** different from Pilo at *p* < 0.05.

**Figure 5.**
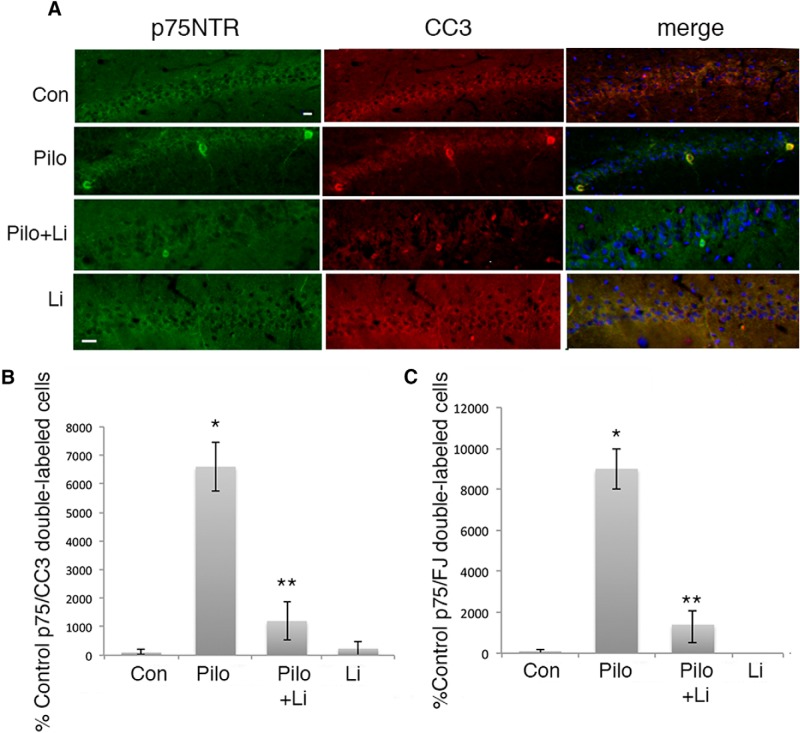
Lithium citrate pretreatment prevents neuronal death in the CA1 region following seizures *in vivo*. Adult male rats were subjected to pilocarpine-induced seizures with or without a 30-min pretreatment with lithium citrate (6 mg/kg) and injections twice daily for 3 d. ***A***, Sections through CA1 showing double labeling with anti-p75^NTR^ and anti-CC3. ***B***, Quantification of p75^NTR^/CC3 double-labeled cells in the CA1 with the different treatments. Cells were counted in every 8th section through the hippocampus. ***C***, Quantification of p75^NTR^/fluorojade-labeled cells in CA1 with the indicated treatments. Scale bars: 10 µm. Quantification in ***B***, ***C*** is expressed as percentage control values which are set at 100%. * different from control at *p* < 0.05, ** different from Pilo at *p* < 0.05.

**Figure 6.**
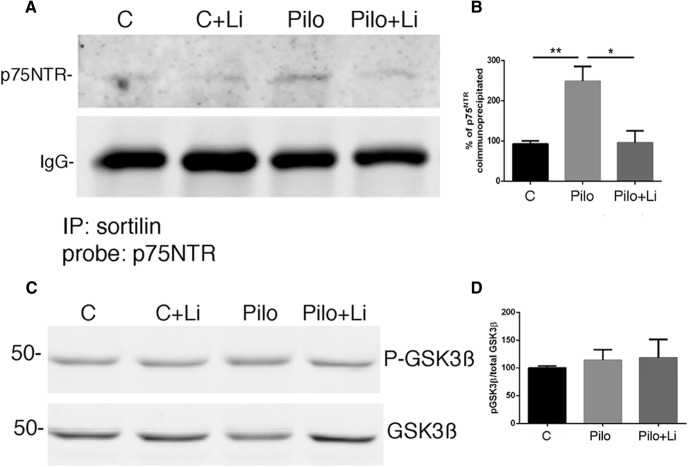
Treatment with lithium citrate reduces p75^NTR^/sortilin interaction *in vivo* after pilocarpine-induced seizures. ***A***, Hippocampal lysates were immunoprecipitated with anti-sortilin and probed for p75^NTR^. ***B***, Quantification of two independent cohorts expressed as percentage control value which is set at 100%; **different from control at *p* < 0.05, *different from pilo at *p* < 0.05. ***C***, Hippocampal lysates were probed for p-GSK3β and total GSK3β. ***D***, Quantification of p-GSK3β relative to total GSK3β shows no effect of lithium citrate treatment *in vivo*.

Pilocarpine-induced seizures elicit neuronal apoptosis in the CA1 and hilus regions of the rat hippocampus at least in part by increasing the level of extracellular proNGF and activating p75^NTR^ apoptotic signaling (Troy et al., 2002; Volosin et al., 2008; Le and Friedman, 2012). Since lithium citrate was able to prevent proNGF binding to the p75^NTR^-sortilin receptor complex, we evaluated whether treatment with a low dose of lithium was able to attenuate neuronal loss in the hippocampus induced by seizures. Adult male rats were pretreated for 30 min with 6 mg/kg lithium citrate given by intraperitoneal injection, and then given pilocarpine to induce seizures. As previously shown, by 3 d after seizure pilocarpine elicited extensive neuronal death in the hilus and CA1 regions of the hippocampus. Pretreatment with lithium citrate followed by twice daily intraperitoneal injections showed significant neuroprotection in both the hilus (Fig. 4) and CA1 (Fig. 5), evaluated by counting cells double-labeled for p75^NTR^ and cleaved caspase-3 (CC3) (Figs. 4*A*,*B*, 5*A*,*B*) or double-labeled for p75^NTR^ and fluorojade C (Figs. 4*C*, 5*C*). The dose of lithium was at least 5-fold lower than doses used in other paradigms of neuroprotection that involved BDNF upregulation (Lauterbach, 2013; Wu et al., 2014) or inhibition of GSK3β (Diniz et al., 2013). We confirmed that this dose of lithium citrate reduced the interaction of p75^NTR^ and sortilin and did not elicit phosphorylation of GSK3β *in vivo* (Fig. 6). The dose was also lower than used in the lithium-pilocarpine model of SE, which uses 127 mg/kg lithium (Hillert et al., 2014) compared with 6 mg/kg used here.

Given the many potential actions of lithium ion, it was important to use the lowest effective dose, and to determine the amount of lithium that gains access to the brain. CSF was collected from the cisterna magna of each animal before being euthanized, and assayed for the level of lithium. Since only 50-70 µl of CSF can be obtained from each animal, samples were pooled from five rats in each of the four treatment categories for analysis of lithium content. Animals with no injected lithium citrate had minimal levels of lithium detected in the CSF (0.84 ng/ml for controls without pilocarpine, and 1.55 ng/ml for animals with pilocarpine). Rats with no pilocarpine, but with 6 mg/kg lithium citrate injected intraperitoneally had 49.34 ng/ml lithium in the CSF, and rats with pilocarpine-induced seizures and injections of 6 mg/kg lithium had 81.1 ng/ml lithium in the CSF, corresponding to 13 µM as an effective neuroprotective dose. This analysis showed that the lithium injected peripherally was able to access the brain, and that this low dose was sufficient to afford neuroprotection.

**Figure 7.**
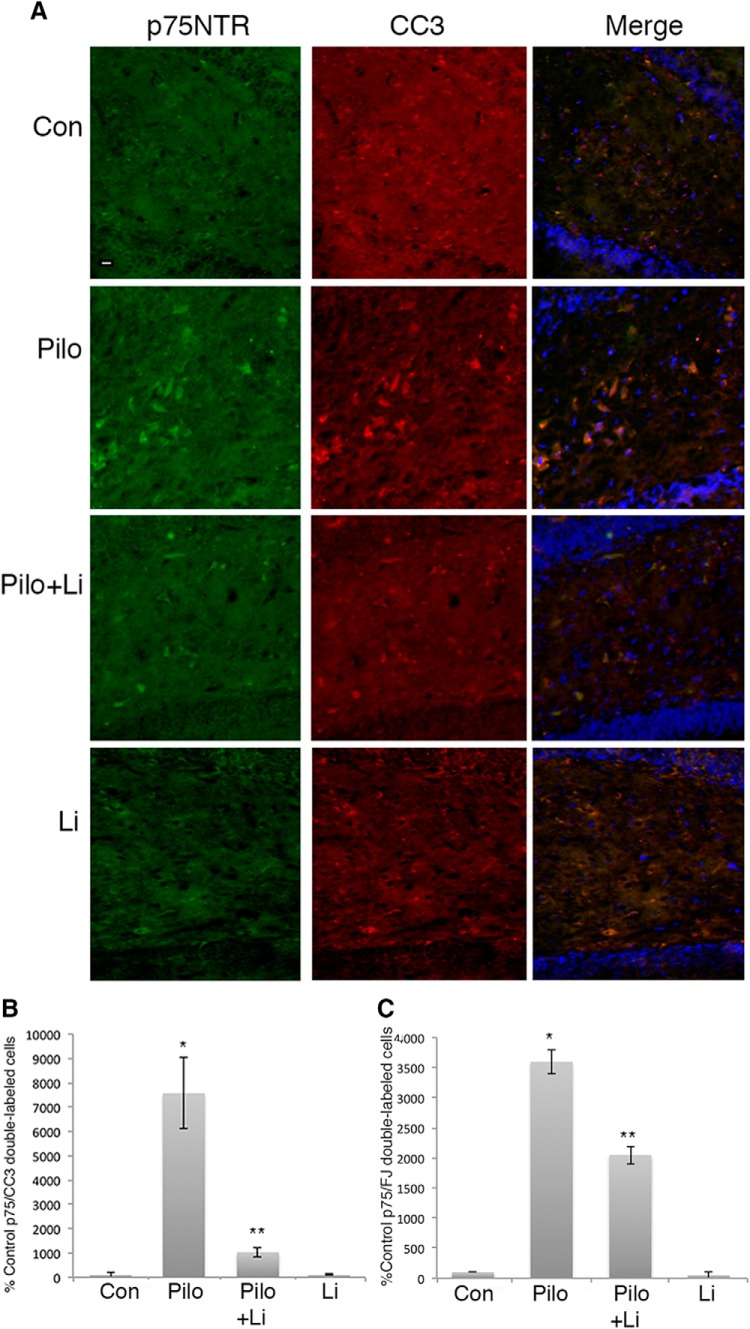
Treatment with lithium citrate after seizures prevents neuronal death in the hilus. Adult male rats were subjected to pilocarpine-induced seizures. Treatment with lithium citrate (6 mg/kg) was initiated after the seizure was terminated and injected twice daily for 3 d. ***A***, Sections through the hilus showing double labeling with anti-p75^NTR^ and anti-CC3. ***B***, Quantification of p75^NTR^/CC3 double-labeled cells in the hilus with the different treatments. Cells were counted in every 8th section through the hippocampus. ***C***, Quantification of p75^NTR^/fluorojade-labeled cells in the hilus with the indicated treatments. Scale bars: 10 µm. Quantification in ***B***, ***C*** is expressed as percentage control values which are set at 100%. * different from control at *p* < 0.05, ** different from Pilo at *p* < 0.05.

**Figure 8.**
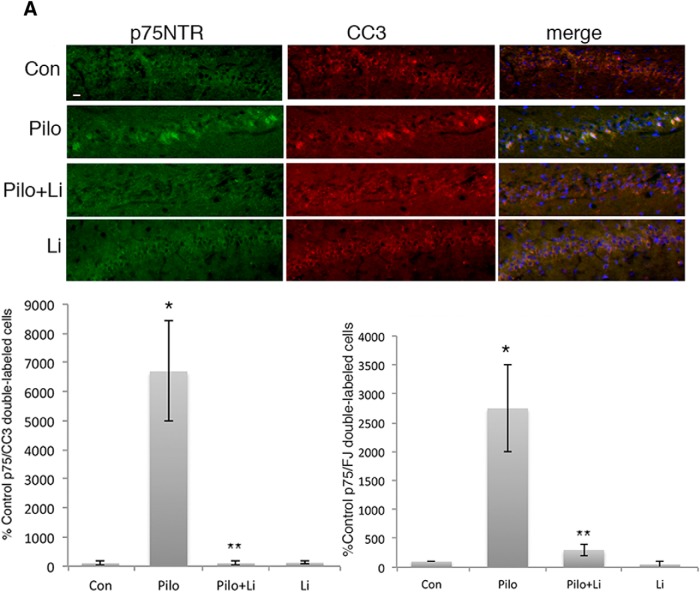
Treatment with lithium citrate after seizures prevents neuronal death in the CA1 region. Adult male rats were subjected to pilocarpine-induced seizures. Treatment with lithium citrate (6 mg/kg) was initiated after the seizure was terminated and injected twice daily for 3 d. ***A***, Sections through CA1 showing double labeling with anti-p75^NTR^ and anti-CC3. ***B***, Quantification of p75^NTR^/CC3 double-labeled cells in the CA1 with the different treatments. Cells were counted in every 8th section through the hippocampus. ***C***, Quantification of p75^NTR^/fluorojade-labeled cells in CA1 with the indicated treatments. Scale bars: 10 µm. Quantification in ***B***, ***C*** is expressed as percentage control values which are set at 100%. * different from control at *p* < 0.05, ** different from Pilo at *p* < 0.05.

To determine whether lithium could also afford neuroprotection if provided after the seizures, adult male rats were treated with lithium citrate delivered intraperitoneally after 1 h of SE, at the time the seizures were terminated with diazepam and phenytoin, followed by twice daily intraperitoneal injections. Even when initially provided after the termination of the seizures, treatment with lithium citrate dramatically reduced the number of apoptotic neurons in both the hilus (Fig. 7) and CA1 (Fig. 8).

## Discussion

### Low dose of lithium citrate is neuroprotective in vivo

Lithium ion has been used as an effective therapy in many models of disease, particularly as a mood-stabilizing drug, as well as to treat brain and spinal cord injury (Young, 2009), where it can protect neurons from death and promote axon sprouting (Dill et al., 2008). Doses of lithium used in these studies range from the micromolar to millimolar range. At these doses, lithium ion has been shown to have many biological activities that can be neuroprotective (Wada et al., 2005; Young, 2009). Among the most well-established functions of lithium ion is the phosphorylation and inhibition of GSK3β and as well as upregulating levels of BDNF and stimulating anti-apoptotic signaling (Rowe and Chuang, 2004; Wada et al., 2005; Young, 2009; Pasquali et al., 2010). Additionally, lithium ion can promote autophagy, which can be neuroprotective, although this was observed at millimolar doses *in vitro* (Motoi et al., 2014; Del Grosso et al., 2016; Liu et al., 2017), and at 50 mg/kg *in vivo* (Liu et al., 2017), doses much higher than those used here. Lithium ion has also been shown to delay disease progression of amyotrophic lateral sclerosis (ALS) in humans and in the mouse G93A model of the disease (Fornai et al., 2008). Since lithium ion can activate so many different neuroprotective signaling pathways, it has many potential therapeutic properties. The effective clinical dose for lithium ion is generally in the range of 0.6-1.0 mM (Young, 2009; Liu et al., 2017). In the current study, we demonstrate a neuroprotective effect of lithium citrate from seizure-induced neuronal loss at a dose several orders of magnitude lower than the established clinical range.

In addition to its neuroprotective function, lithium is often used together with pilocarpine to generate a model of epileptic seizures, however the dose of lithium used in that model is a significantly higher [3 mEq (Jope et al., 1986) or 127 mg/kg (Hillert et al., 2014)] than the protective dose used here (6 mg/kg).

In injury models of TLE, severe continuous seizures defined as SE lead to increased expression and stabilization of proNGF, which binds with selective high affinity to the p75^NTR^-sortilin receptor complex and elicits apoptosis (Friedman, 2010). p75^NTR^ is widely expressed in the CNS during development, but in the adult hippocampus, this receptor is expressed primarily following injury. Previous studies have demonstrated that proNGF and p75^NTR^ play a major role in mediating neuronal death after SE (Troy et al., 1997; Volosin et al., 2008; Le and Friedman, 2012), we anticipated that disrupting this association may prevent neuronal death after SE. In these studies, pilocarpine was used to induce seizures. Animals treated with pilocarpine showed increased expression of p75^NTR^ colocalized with CC3, the main executor protein in the apoptotic pathway, which was maximal by 3 d after the seizures in the CA1 and dentate hilus regions, consistent with previous studies (Roux et al., 1999; Troy et al., 2002). Fluorojade C was additionally used to identify dying neurons, and also demonstrated increased double-labeling with p75^NTR^ after pilocarpine treatment. Pretreatment and twice daily injections with the low dose of lithium (6 mg/kg) resulted in decreased p75^NTR^ expression and reduced neuronal death compared to pilocarpine alone, evaluated by CC3 and fluorojade C labeling, in both the CA1 and hilus. Moreover, this treatment elicited a reduction in the association of the p75^NTR^-sortilin receptor complex *in vivo*. The amount of lithium measured in the CSF corresponded to 13 µM. As is common for drugs administered *in vivo*, this amount of lithium is higher than the lowest protective dose shown *in vitro*, but is well below the usual clinical dose (Young, 2009). These results demonstrated that a low dose of lithium can afford neuroprotection from seizure-induced neuronal death. The identification of lithium ion as an inhibitor of proNGF binding to its p75^NTR^-sortilin receptor complex suggested that this may represent a novel mechanism for neuroprotection.

Since there is a time lag after seizures for the induction of p75^NTR^ and proNGF to occur before the time of maximal neuronal loss, we evaluated whether lithium citrate would be neuroprotective if provided after SE rather than before the seizures. Rats were therefore given the first dose of lithium when the seizures were terminated with diazepam. In these experiments lithium citrate still afforded significant neuroprotection and reduced cell death in the CA1 and hilus, indicating that lithium citrate can be given after seizures and still prevent neuronal loss. The extent of the time window for neuroprotection after seizures remains to be determined, however being able to provide a neuroprotective agent after the seizure event may be of potential therapeutic value.

We show that lithium citrate has neuroprotective effects *in vivo* following seizures using doses well below the clinical range used to treat other disorders. The pattern of damage seen in animals treated with both pilocarpine and lithium citrate illustrates that the drug disrupts the well-described pattern of damage following SE. Measurement of lithium levels in the CSF demonstrated that peripherally-injected lithium citrate at this low dose (6 mg/kg) gained access to the brain, and provided a significant protective effect on hippocampal neurons following SE *in vivo*.

### Mechanisms of neuroprotection by lithium

Cultured hippocampal neurons were used to investigate mechanisms by which lithium ion could afford neuroprotection, since Li^+^ is known to prevent neuronal death through multiple pathways (Pasquali et al., 2010). Lithium ion can induce phosphorylation and inactivation of GSK3β, a Ser/Thr kinase that is abundant in CNS neurons and promotes cell death by blocking the nuclear translocation of beta catenin. Lithium ion can also induce an increase in BDNF levels (Fukumoto et al., 2001; Wu et al., 2014), which can promote neuronal survival by activating the TrkB receptor and downstream Akt and Erk signaling. To assess whether lithium citrate may protect neurons by phosphorylation and inactivation of GSK3β or by upregulating BDNF expression, we compared the doses of lithium citrate required for protection from proNGF-induced apoptosis with those required for activation of GSK3β in cultured neurons and *in vivo*. We found that the doses of lithium that protected neurons from proNGF-induced death *in vitro*, or SE-induced death *in vivo*, were insufficient to elicit phosphorylation of GSK3β or upregulation of BDNF, suggesting that neither phosphorylation of GSK3β nor induction of BDNF was responsible for preventing neuronal death in these paradigms.

### Lithium citrate prevents association and internalization of the p75^NTR^-sortilin receptor complex

The original identification of lithium as a potential inhibitor of proNGF-induced neuronal death was based on its ability to prevent binding to the receptor complex, suggesting that the mechanism of protection might be at the level of the membrane receptors. We investigated whether lithium at the neuroprotective nanomolar concentration was effective in blocking either the association of p75^NTR^ with sortilin, or the internalization of the two receptors following proNGF treatment in culture. Coimmunoprecipitation analysis demonstrated that lithium citrate decreased the association of p75^NTR^ and sortilin both *in vitro* and *in vivo*, thereby reducing formation of the requisite receptor complex for proNGF to induce apoptosis. Using surface biotinylation assays to track the internalization of membrane receptors, we also found that lithium reduced the internalization of p75^NTR^ and sortilin into the neurons. These data suggest that the neuroprotective effect of lithium citrate at these low doses may be through disrupting the association and internalization of p75^NTR^ and sortilin and thereby preventing proNGF apoptotic signaling. Although lithium ion can exert neuroprotection through multiple mechanisms, some of the well-established pathways of lithium actions were not activated by this treatment paradigm. Other receptors and pathways are likely to be affected by the lithium citrate treatment as well, however preventing the activation of the p75^NTR^-sortilin receptor complex may contribute to the neuroprotective effects.

A previous study demonstrated that the binding of proNGF to the p75^NTR^-sortilin receptor complex is stabilized in the presence of calcium (Feng et al., 2010). It is well established that the prodomain of proNGF is intrinsically disordered, and thus may adopt distinct, transient conformational changes in response to environmental perturbations. Indeed, addition of calcium at physiologic levels enhanced the interaction of the prodomain region with sortilin, and stabilized the formation of a ∼600 kDa proNGF/sortilin/p75 complex. In contrast, calcium chelation significantly reduced the interaction of proNGF with its receptors. One possible mechanism for the actions of lithium ion may be to alter the conformation of the prodomain region of proNGF, possibly leading to the attenuation of the proNGF-induced p75^NTR^-sortilin association and/or internalization, and suggesting a novel mechanism for neuroprotection by lithium ion.

In summary, we demonstrated in this study that low doses of lithium citrate were able to prevent proNGF-induced apoptosis of cultured hippocampal neurons, and provided significant neuroprotection from seizure-induced neuronal loss, even when provided after termination of the seizures. These data suggest a novel therapeutic use for lithium ion to provide neuroprotection from seizures at nanomolar doses, well below the dose currently in clinical use, thereby minimizing potential side effects from the multiple actions of lithium ion that occur at higher micromolar doses.

## Synthesis

Reviewing Editor: Leonidas Stefanis, Biomedical Research Foundation of the Academy of Athens Decisions are customarily a result of the Reviewing Editor and the peer reviewers coming together and discussing their recommendations until a consensus is reached. When revisions are invited, a fact-based synthesis statement explaining their decision and outlining what is needed to prepare a revision will be listed below. The following reviewer(s) agreed to reveal their identity: Lloyd Greene, Bruce Carter.

Both reviewers fell that the main observation of low lithium ion concentrations impairing pro-NGF signaling is well substantiated and of interest. However, the manuscript needs to be revised according to the detailed comments of the reviewers below, with particular attention to:

1. Rearranging the order of presentation of the results, as suggested by Reviewer 1.

2. Providing the data of the screening.

3. Quantifying all data in Figure 5.

4. Providing in vivo data on effects on GSK3beta.

5. Examining whether lithium ion may have more general effects on formation of receptor complexes and/or their internalization.

6. Adding a note of caution in the discussion regarding the in vivo protective effects of lithium ion, which may be due to many of its other actions. It would be particularly important if, along these lines, an effect on the p75/sortilin complex were demonstrated in the in vivo model.

7. Streamlining and focusing the discussion.

Reviewer #1:

This ms provides data that a relatively low concentration of lithium ion reduces association of pro-NGF with p75 and sortilin and interferes with internalization of the pro-NGF-p75-sortilin complex. It also provides data that lithium ion protects cultured neurons from pro-NGF in vitro and neurons in an in vivo model of epilepsy in which neuron death requires the actions of pro-NGF. The authors provide evidence that the protective actions are not due to several other potential actions of lithium ion treatment and argue that they are due to lithium ion's effect on the pro-NGF-p75-sortilin complex.

As can be seen below, I've a number of reservations about this manuscript that need to be addressed, so let me state up front that despite this, I think that the findings are of considerable potential interest and should ultimately be published. Irrespective of the flaws here, the observation that low lithium ion concentrations appear to affect pro-NGF signaling should promote further work in this area and could be clinically relevant.

1. The paper refers to "lithium" on many occasions. Lithium is an element. What the authors mean is lithium ion or Li+ or lithium citrate.

2. Lim et al referenced in the introduction, is not in the reference list. Same with Endo et al. The referring sentence states "Lithium can inhibit apoptosis by increasing Akt, and by phosphorylating and inactivating GSK-3beta." Does lithium ion really increase Akt and phosphorylate GSK-3beta, or does it indirectly affect their activity and state of phosphorylation?

3. It would be useful if the paper were to list the 15 candidate compounds identified in the initial screen. Perhaps I missed it, but it also would be important to know the total number of candidates that were tested in the initial screen. Finally, it would be important to show the Li ion results from the screen rather than just state that it blocked uptake without showing any data.

4. It seems awkward to start the figures with an in vivo experiment involving neuroprotection without first presenting data that demonstrate that lithium ion actually prevents the uptake of pro-NGF and that it interferes with the association of pro-NGF with p75/sortilin. The findings of the initial screen could be due to a general blockade of lithium citrate on protein uptake/transport and could have nothing to do with pro-NGF association with its receptor complex. Likewise, at this point of the manuscript, one could well imagine that the actions of lithium citrate involve mechanisms totally unrelated to pro-NGF and its interaction with its receptors. I'd suggest starting with the material in Figs 6 and 7, followed by the in vitro work and then the in vivo study. The organization of the data should be up to the authors, but in my view, the data do not flow well as currently presented.

5. In the second paragraph of results, the authors state that their rationale for carrying out the in vivo pilocarpine model was that Li ion was able to prevent proNGF binding to p75/sortilin. However, at this point in the paper, this has not been shown. The only data mentioned at this point in the ms is that Li ion blocks uptake of pro-NGF. This issue reinforces the point above about organization of the paper.

6. The paper states" The dose was also lower....compared with the 6 mg/kg used here." The model that is being compared with the present work needs to be referenced. The paper is also a little vague about prior work that has shown protection by lithium ion in the pilocarpine model. A pubmed search reveals a paper by Hong et al (Korean J Physiol. Pharm. Jan 2017) that showed protection in this model by lithium ion given at a single dose of 80 mg/kg in mice.

7. Fig 1-4. The text and legend should indicate the time after pilocarpine treatment that the brains were harvested. The methods indicate 3 days, but this is not clearly spelled out. Also, the methods indicate that the animals received lithium citrate injections every 12 hours. It is unclear whether this was for both the pre- and post-seizure treatments. Also, it is unclear why repeated doses were given and how important this was for protection. The information about repeated treatment with lithium citrate should also be indicated in the text and legend as it is germane to the study and its interpretation.

8. Is the fact that lithium ion given 1 hr after seizure was effective surprising given that the major effects of cell death are seen 3 days later? At what time in this model would one expect to see elevation of pro-NGF and its interaction with elevated p75/sortilin?

9. The text keeps referring to the lithium ion concentration used and detected in the CSF as a "low dose". However, the 13-micromolar concentration in the CSF is 2 orders of magnitude higher than the lowest effective in vitro dose reported in the paper. Also the 13 micromolar CSF level appears to have been measured 12 hours after the last lithium ion treatment. The highest dose in the CSF reached during the study (presumably soon after injection) is not given nor is this considered. Thus, on the basis of the data that are presented, it is difficult to accept the conclusion that the in vivo protective effects of lithium ion are necessarily due to interference with pro-NGF-receptor interaction. While this could be the case, alternative explanations are possible. A more convincing experiment would be to show that as applied in these experiments lithium ion blocks the pro-NGF-receptor interaction/uptake/activation in brain.

10. It would be useful to quantify and normalize the blot data in Fig 5B, especially since the level of total gsk3b appear to increase with increasing lithium ion.

11. The 6-bromoindirubin-3'-oxime experiment that was not shown is said to have resulted in "toxicity independent of pro-NGF (not shown)". If the drug was toxic, how could one interpret the study as representing a "different method of inactivating GSK3beta"?

12. The paper suggests that lithium ion interferes with association of pro-NGF with the p75-sortilin receptor and that this in turn affects uptake of the complex. However, the data in Fig 6 indicate a rather mild effect of lithium ion on the capacity of pro-NGF to affect p75-sortilin interaction while in contrast, the data in Fig 7 suggest a much greater effect of lithium ion on internalization of the complex. This raises the question of whether the observed effects of lithium ion on survival are due mainly to effects on formation of the receptor complex or to its effects on internalization. That is, the effect of lithium ion on internalization could be at least partly independent of its actions on formation of the complex. These points ought to be considered in the discussion.

13. A major question that is not addressed in the paper is whether the described effects of lithium ion are specific to the pro-NGF/p75/sortilin complex. That is, does lithium ion have broader effects on formation of surface receptor complexes and/or on their internalization?

14. The discussion is largely a summary of the experiments that were done and could be greatly shortened. The discussion would be more interesting if it acknowledged and addressed some of the points raised above.

Reviewer #2:

This manuscript reports on a novel mechanism by which lithium (Li+) protects neurons from apoptosis induced by seizures, which is particularly interesting given that Li+ combined with pilocarpine is a well established model for inducing seizures and neuronal damage. The authors emphasize that the protective effects of Li+ are at a much lower level that those used to augment the toxic effects of pilocarpine-mediated seizures. Their results indicate that Li+ is protective through inhibiting the formation of a proNGF-p75-Sortilin complex and preventing internalization of p75, which normally leads to neuronal death. Although Li+ affects a number of signaling pathways, including inhibition of GSK3b and up regulation of BDNF, the authors provide data indicating that the dose of Li+ they are using is far below that needed to affect these signals. The findings demonstrate a new therapeutic mechanism by which Li+ can act, which is novel and interesting; however, the link between the protective effects of Li+ in the seizure model and p75 signaling are only correlative at this point. The authors could strengthen their findings and provide data supporting a direct link by inducing seizures in p75-/- mice treated with or without Li+ at the low dose they use here. Previous work by Troy et al (JBC, 2002) demonstrated a reduction in neuronal apoptosis following pilocarpine-induced seizures in mice lacking p75, but there was still a significant degree of neuron death. If the hypothesis of the current study is correct, then Li+, at these low doses, should have no additional protective effects in the p75-/- mice. Given that this would involve significant additional experimentation, the authors need to at least acknowledge that their findings in vivo are only correlative and they can't rule out other protective mechanisms by which Li+ may be working. Li+ affects a very wide range of signaling pathways, beyond GSK3b and BDNF, e.g. modulation of phosphoinositide metabolism and G-protein coupled receptor function. Therefore, the in vivo results presented may be mediated by other mechanisms despite the in vitro evidence that Li+ affects proNGF-p75 signaling.

The western blots in Fig. 5B and C should be quantified to confirm the authors claim that these pathways were not affected by this dose of Li+.

To further substantiate the lack of GSK3b involvement in the protective effects of Li+ in the seizure model, the authors should blot the brain lysates from the animals treated with and without Li+ for phospho-GSK3b.

## Author Response

November 9, 2017

Dear Dr. Stefanis,

We thank the reviewers for their helpful comments. We have incorporated the suggestions and changes into the manuscript and respond to the reviewers' comments in italics below. All animal studies were conducted using the National Institutes of Health guidelines for the ethical treatment of animals with approval of the Rutgers Institutional Animal Care and Facilities Committee.

Reviewer 1.

1. The paper refers to “lithium” on many occasions. Lithium is an element. What the authors mean is lithium ion or Li+ or lithium citrate.

We have revised the terminology throughout the manuscript to indicate either lithium citrate or lithium ion.

2. Lim et al referenced in the introduction, is not in the reference list. Same with Endo et al. The referring sentence states “Lithium can inhibit apoptosis by increasing Akt, and by phosphorylating and inactivating GSK-3beta.” Does lithium ion really increase Akt and phosphorylate GSK-3beta, or does it indirectly affect their activity and state of phosphorylation?

These references were in error and we apologize for that. We now include references to papers showing that 1-10 mM lithium ion exerts neuroprotection via Akt activation and increasing phosphorylation of GSK3ß.

3. It would be useful if the paper were to list the 15 candidate compounds identified in the initial screen. Perhaps I missed it, but it also would be important to know the total number of candidates that were tested in the initial screen. Finally, it would be important to show the Li ion results from the screen rather than just state that it blocked uptake without showing any data.

We have indicated this information in the manuscript. Specifically, 2560 compounds were tested and a list of the 15 compounds which exhibited a reduction of proNGF uptake by 65% or more without including toxicity of 15% or greater, and have been used in humans are listed in Table 1. Data on inhibition of proNGF uptake are provided.

4-5. Organization of the manuscript.

We agree with the reviewer's comments about the organization of the manuscript, and have reorganized it with the in vitro data before the in vivo data.

6. The paper states“ The dose was also lower....compared with the 6 mg/kg used here.” The model that is being compared with the present work needs to be referenced. The paper is also a little vague about prior work that has shown protection by lithium ion in the pilocarpine model. A pubmed search reveals a paper by Hong et al (Korean J Physiol. Pharm. Jan 2017) that showed protection in this model by lithium ion given at a single dose of 80 mg/kg in mice.

The lithium-pilocarpine is an established model for generating seizures and eliciting neuronal loss, the point being made is that that model uses 127 mg/kg of lithium, which was referenced in the discussion (Hillert et al, 2014). We apologize for leaving out that reference at this place in the manuscript, it is now included with that statement.

7. Fig 1-4. The text and legend should indicate the time after pilocarpine treatment that the brains were harvested. The methods indicate 3 days, but this is not clearly spelled out. Also, the methods indicate that the animals received lithium citrate injections every 12 hours. It is unclear whether this was for both the pre- and post-seizure treatments. Also, it is unclear why repeated doses were given and how important this was for protection. The information about repeated treatment with lithium citrate should also be indicated in the text and legend as it is germane to the study and its interpretation.

The text and figure legends now explicitly state the time point was 3 days after seizures, which is when we see maximal cell death. The lithium citrate injections were given twice a day, and this is standard for lithium treatments in clinical settings. The pharmacokinetics of lithium have been well described and multiple daily dosing are considered to better maintain more constant plasma lithium concentration.

8. Is the fact that lithium ion given 1 hr after seizure was effective surprising given that the major effects of cell death are seen 3 days later? At what time in this model would one expect to see elevation of pro-NGF and its interaction with elevated p75/sortilin?

The fact that the lithium citrate was effective when given after the seizures is important for several reasons. Since there is a time lag during which the levels of p75 and proNGF are being elevated it confirms that neuroprotective drugs provided during this time lag may still be effective. Moreover, in considering potential therapeutic aspects, since one cannot predict when seizures may occur to give drugs prophylactically, this demonstrates that lithium citrate can be provided after the seizure and still provide neuroprotection.

9. The text keeps referring to the lithium ion concentration used and detected in the CSF as a “low dose”. However, the 13-micromolar concentration in the CSF is 2 orders of magnitude higher than the lowest effective in vitro dose reported in the paper. Also, the 13 micromolar CSF level appears to have been measured 12 hours after the last lithium ion treatment. The highest dose in the CSF reached during the study (presumably soon after injection) is not given nor is this considered. Thus, on the basis of the data that are presented, it is difficult to accept the conclusion that the in vivo protective effects of lithium ion are necessarily due to interference with pro-NGF-receptor interaction. While this could be the case, alternative explanations are possible. A more convincing experiment would be to show that as applied in these experiments lithium ion blocks the pro-NGF-receptor interaction/uptake/activation in brain.

The low dose is in comparison to doses currently used in vivo in animals or clinically in humans, which is in the high micromolar to millimolar range, and that sentence in the Discussion has been re-phrased. Since clinically, one needs to know how much lithium is there in the troughs between treatment times to maintain a minimal level of lithium in the brain. With respect to other potential mechanisms for lithium actions, this is certainly true and discussed in the Discussion. With respect to the final comment, we now show that this dose reduced the p75sortilin interaction in vivo without inducing phosphorylation of GSK3ß (new figure 6).

10. It would be useful to quantify and normalize the blot data in Fig 5B, especially since the level of total gsk3b appear to increase with increasing lithium ion.

A graph showing the quantification is now included with the figure. The point we are making is that at these nanomolar to micromolar doses, which is where we see neuroprotection, no significant GSK3ß phosphorylation was observed relative to the amount of total GSK3ß, consistent with the literature which shows millimolar doses of lithium used to phosphorylate GSK3ß.

11. The 6-bromoindirubin-3'-oxime experiment that was not shown is said to have resulted in “toxicity independent of pro-NGF (not shown)”. If the drug was toxic, how could one interpret the study as representing a “different method of inactivating GSK3beta”?

We used BIO, which inactivates GSK3ß independent of the actions of lithium, to confirm that inactivating GSK3ß was not the mechanism by which lithium citrate was protecting neurons from proNGF-induced death. In a dose-response analysis, none of the doses of BIO protected from proNGF-induced death, and at high doses this drug caused toxicity. Since the toxicity of BIO at high dose is not relevant to the point being made, that phrase has been removed.

12. The paper suggests that lithium ion interferes with association of pro-NGF with the p75sortilin receptor and that this in turn affects uptake of the complex. However, the data in Fig 6 indicate a rather mild effect of lithium ion on the capacity of pro-NGF to affect p75-sortilin interaction while in contrast, the data in Fig 7 suggest a much greater effect of lithium ion on internalization of the complex. This raises the question of whether the observed effects of lithium ion on survival are due mainly to effects on formation of the receptor complex or to its effects on internalization. That is, the effect of lithium ion on internalization could be at least partly independent of its actions on formation of the complex. These points ought to be considered in the discussion.

The reviewer correctly points out that effects on internalization of the receptor complex may be independent on the effects on receptor association. We agree with this, and in the paper we indicate only that lithium ion interferes with both processes, receptor association and internalization, without indicating that one is dependent on the other.

13. A major question that is not addressed in the paper is whether the described effects of lithium ion are specific to the pro-NGF/p75/sortilin complex. That is, does lithium ion have broader effects on formation of surface receptor complexes and/or on their internalization?

Certainly the effects of lithium ion may have broader effects than on the proNGF-p75/sortilin interactions. This is acknowledged in the Discussion, however we can only speak to specific receptor complexes that we have analyzed.

14. The discussion is largely a summary of the experiments that were done and could be greatly shortened. The discussion would be more interesting if it acknowledged and addressed some of the points raised above.

The Discussion has been revised as suggested by the reviewer.

Reviewer 2:

We acknowledge the reviewer's point that lithium can protect via many mechanisms, and we have emphasized this now in the Discussion.

The western blots in former figure 5B and C (now figure 1B and C) now show the quantification, and the point we wish to emphasize is that at doses that are neuroprotective, no GSK3ß phosphorylation or BDNF induction is evident.

We have now generated brain lysates from animals with and without pilocarpine-induced seizures treated with vehicle or lithium citrate. We show that this low dose has no effect on PGSK3ß in vivo. The same samples were analyzed by co-IP for p75-sortilin interactions, and show a decreased association, confirming the in vitro results that the low dose of lithium citrate can disrupt the receptor complex without affecting P-GSK3ß. Those data are now included as new figure 6.

Sincerely,

Wilma Friedman, Ph.D.

Professor
